# Supplementary material for: Acute Inflammation Is a Predisposing Factor for Weight Gain and Insulin Resistance
Source: Pharmaceutics. 2022 Mar 11;14(3):623. doi: 10.3390/pharmaceutics14030623 (PMC8954490; doi:10.3390/pharmaceutics14030623)
Supplement: Supplementary file 1 [file pharmaceutics-14-00623-s001.zip › pharmaceutics-1586354-supplementary.pdf]

Figure S1

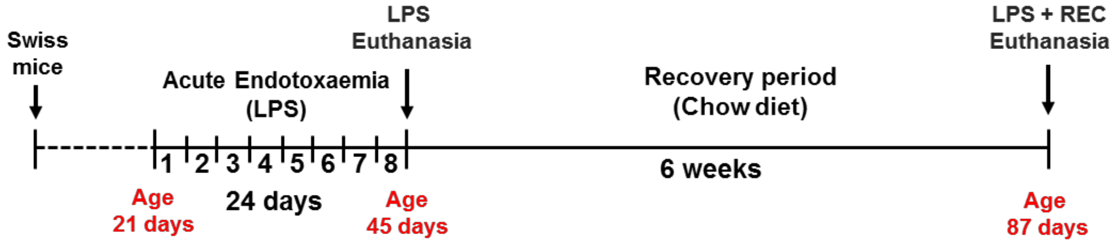

Figure S1a. Acute endotoxemia followed by recovery period under chow diet (LPS + REC). The method of multiple inductions of acute endotoxemia comprises intraperitoneal administration of 8 consecutive injections (every 3 days) of LPS 10 mg/kg (Lipopolysaccharides from *Escherichia coli* 026:B6, Sigma-Aldrich®, St. Louis, MO, USA), in saline (NaCl 0,9%), starting at weaning (21 days of age) with end at 45 days of age of the animal, followed by a recovery period of 6 weeks under chow diet. For acute endotoxemia experiments, mice were randomly assigned into 2 different groups: the Control group and the LPS group, with euthanasia occurring after the last acute phase period or after 6 weeks from the last acute phase induction (recovery period).

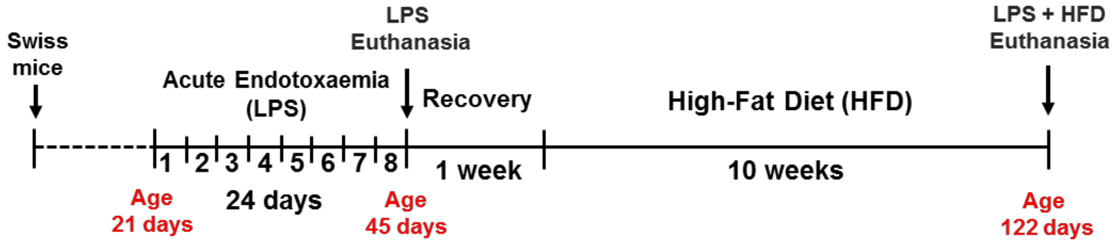

Figure S1b. Acute endotoxemia followed by High-Fat Diet (HFD) (LPS + HFD). For acute endotoxemia followed by 10 weeks on a high-fat diet (LPS+HFD) experiments, the animals were randomly assigned into 2 different groups: HFD group and LPS+HFD group. The HFD mice were submitted to a HFD for 10 weeks starting concurrently with the LPS+HFD group. The LPS+HFD mice were underwent to multiple inductions of acute endotoxemia followed by 1 week of recovery period in standard chow diet plus 10 weeks on a HFD.

Figure S2

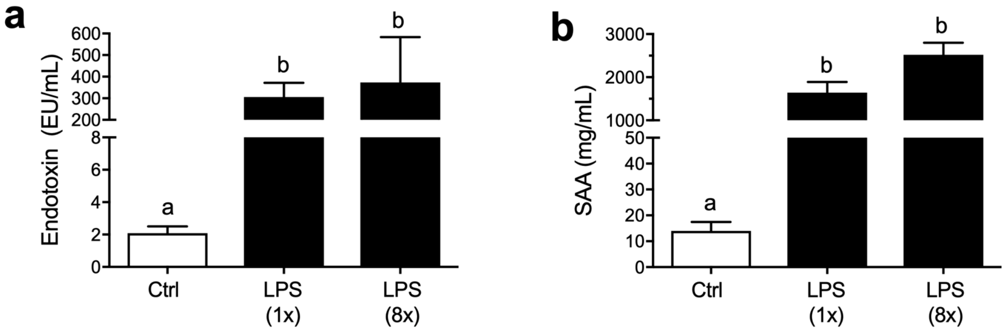

Figure S2. Serum endotoxin and SAA levels after LPS challenges. Swiss Webster mice were submitted to intraperitoneal administration of 8 consecutive doses of 10 mg/kg LPS, every 3 days. **(a)** Endotoxin and **(b)** SAA concentration in serum. For all variables with the same letter, the difference between the means is not statistically significant. Where two variables have different letters, they are significantly different ($p < 0.05$). Data are means ± SEM from 6 mice per group.

Table S1. SAA profile during acute endotoxemia

	SAA (µg/mL)	
Time (hours)	Control group	LPS group
0 h	16.8 ± 8.2	15.4 ± 6.8
6 h	21.7 ± 6.8	986.4 ± 75.8***
12 h	25.4 ± 13.5	1527.0 ± 193.6***
24 h	18.2 ± 10.7	888.4 ± 141.0**
48 h	7.1 ± 2.3	125.4 ± 40.1**
72 h	17.5 ± 8.7	29.6 ± 11.7

Data are means ± SD from 3 mice per group
(***p*<0.01, ****p*<0.001, between groups, as indicated)

Table S1. SAA profile during acute endotoxemia. SAA was quantified in serum after 6, 12, 24, 48 and 72 hours of LPS-treatment (10 mg/kg).

Table S2. Experimental diet composition

Ingredients (g/Kg)	Chow diet[a] 16.7 kJ/g	High-fat diet 23.2 kJ/g
Sucrose	100	133.56
Casein	120	186.98
Corn oil	80	53.42
Lard	-	300
Cellulose	50	66.78
Mineral Mix (Rhoster®)	35	46.74
Vitamin Mix (Rhoster®)	10	13.36
DL-Methionine[b]	1.8	2.4
Choline Bitartrate	2.5	3.34
Tert-butylhydroquinone	0.01	0.04
Corn starch	600.69	193.38

[a]According to AIN-93M.
[b]2-amino-4-methylsulfanylbutanoic acid.

Table S3. Oligos used in all quantitative PCR assays

Primer (gene / protein)	Forward	Reverse
Saa1.1/2.1 (SAA1 / SAA2)	5'-AGA CAA ATA CTT CCA TGC TCG G-3'	5'-CAT CAC TGA TTT TCT CAG CAG C-3'
Tlr2 (TLR-2)	5'-CAG CTG GAG AAC TCT GAC CC-3'	5'-CAA AGA GCC TGA AGT GGG AG-3'
Tlr4 (TLR-4)	5'-TCA TGG CAC TGT TCT TCT CCT-3'	5'-CAT CAG GGA CTT TGC TGA GTT-3'
Cd14 (CD14)	5'-GCG AGC TAG ACG AGG AAA GT-3'	5'-CAC GCT TTA GAA GGT ATT CCA G-3'
Gapdh (GAPDH)	5'-TGG CAA AGT GGA GAT TGT TGC C-3'	5'-AAG ATG GTG ATG GGC TTC CCG-3'
